# Extracting Signs and Symptoms of Hypertensive Disorders in Pregnancy from Clinical Notes Using Natural Language Processing

**DOI:** 10.1007/s10995-026-04275-y

**Published:** 2026-05-20

**Authors:** Jihye Kim Scroggins, Zhihong Zhang, Ismael I. Hulchafo, Maxim Topaz, Veronica Barcelona

**Affiliations:** 1 School of Nursing, University of North Carolina at Chapel Hill, 120 Medical Dr, Chapel Hill, NC 27514 USA; 2 Data Science Institute, Columbia University, New York, NY USA; 3 School of Nursing, Columbia University, New York, NY USA

**Keywords:** Natural language processing, Hypertensive disorders, Symptom science, Health disparities, Maternal morbidity

## Abstract

**Purpose:**

Hypertensive disorders in pregnancy (HDP) affect 16% of births in the United States. In this pilot study, we conducted a preliminary evaluation of natural language processing (NLP) in extracting signs and symptoms (SS) of HDP from clinical notes within electronic health records (EHRs).

**Methods:**

This retrospective observational pilot study used EHR data from patients admitted for labor and birth (*N* = 83,003 clinical notes from 17,775 patients). Four SS categories were extracted: elevated blood pressure, neurological, renal, and hepatic/hematologic. Five machine learning models and ClinicalBERT were trained and tested using five-fold cross-validation. The best-performing model was applied to the full dataset. Bivariate analyses were performed to examine (1) differences in HDP diagnoses based on ICD-10 codes (gestational hypertension, preeclampsia, and eclampsia) by SS documentation and (2) differences in SS documentation by patient race and ethnicity.

**Results:**

XGBoost demonstrated the highest macro-average F1-score (0.75). Elevated blood pressure showed the highest F1-score (0.87), followed by neurological SS (0.77). In the full dataset, 24.3% of clinical notes and 42.3% of patients had documentation of at least one SS category. A higher proportion of HDP diagnoses was observed with an increased number of SS categories documented (*p* < .001). A higher proportion of non-Hispanic Black patients had documentation of SS across all categories.

**Conclusion:**

NLP can extract SS with moderate accuracy, supporting feasibility for larger-scale extraction. Findings also highlight differences in SS documentation by patient race and ethnicity. Future research is needed to improve NLP performance, including expanding annotated data.

**Supplementary Information:**

The online version contains supplementary material available at 10.1007/s10995-026-04275-y.

## Significance

What is Already Known

What This Study Adds

Hypertensive disorders in pregnancy (HDP) affect approximately 16% of births in the United States. Previous studies have used natural language processing (NLP) to extract signs and symptoms of various health conditions, such as heart failure and cancer. However, it is underused in perinatal populations.

This pilot study used NLP to extract signs and symptoms of HDP from narrative clinical notes with moderate performance, supporting methodological feasibility for larger-scale extraction. Findings also highlight differences in documentation patterns across racial and ethnic groups. Future research is needed to improve NLP performance, including expanding annotated data.

**Supplementary Information:**

The online version contains supplementary material available at 10.1007/s10995-026-04275-y.

## Introduction

Hypertensive disorders in pregnancy (HDP), including chronic hypertension, gestational hypertension, preeclampsia, eclampsia, and unspecified maternal hypertension, affect approximately 16% of births in the United States (Ford et al., 2022). HDP increase the risk of adverse perinatal outcomes and long-term cardiovascular diseases (Garovic et al., 2022). For example, preeclampsia has been associated with higher odds of preterm birth and low birth weight (Bromfield et al., 2023). Similarly, gestational hypertension has been associated with a higher risk of developing cardiovascular disease later in life (Riise et al., 2018). Thus, timely identification and treatment of HDP are crucial to prevent potential adverse health outcomes during and beyond the perinatal period (Chourdakis et al., 2021). Importantly, HDP disproportionately affect Black and Hispanic patients. HDP are among the top five causes of pregnancy-related deaths for non-Hispanic Black patients (Trost et al., 2022). Both Black and Hispanic patients have higher odds of preeclampsia or eclampsia at the time of birth than white patients (Minhas et al., 2021). Similarly, eclampsia rates are higher among non-Hispanic Black and Hispanic patients than non-Hispanic white patients (Admon et al., 2018).

Electronic health records (EHRs) contain a large volume of clinically relevant data, such as signs and symptoms (SS), which are important for understanding and characterizing health conditions. Free-text narratives documented in unstructured clinical notes offer a promising, complementary data source for capturing SS. However, this information is often underutilized due to challenges in data extraction and processing using traditional analytic techniques (Sim et al., 2023). Natural language processing (NLP) is a subfield of computer science that enables the extraction of clinically relevant information from large text data (Topaz et al., 2019).

**Fig. 1:**
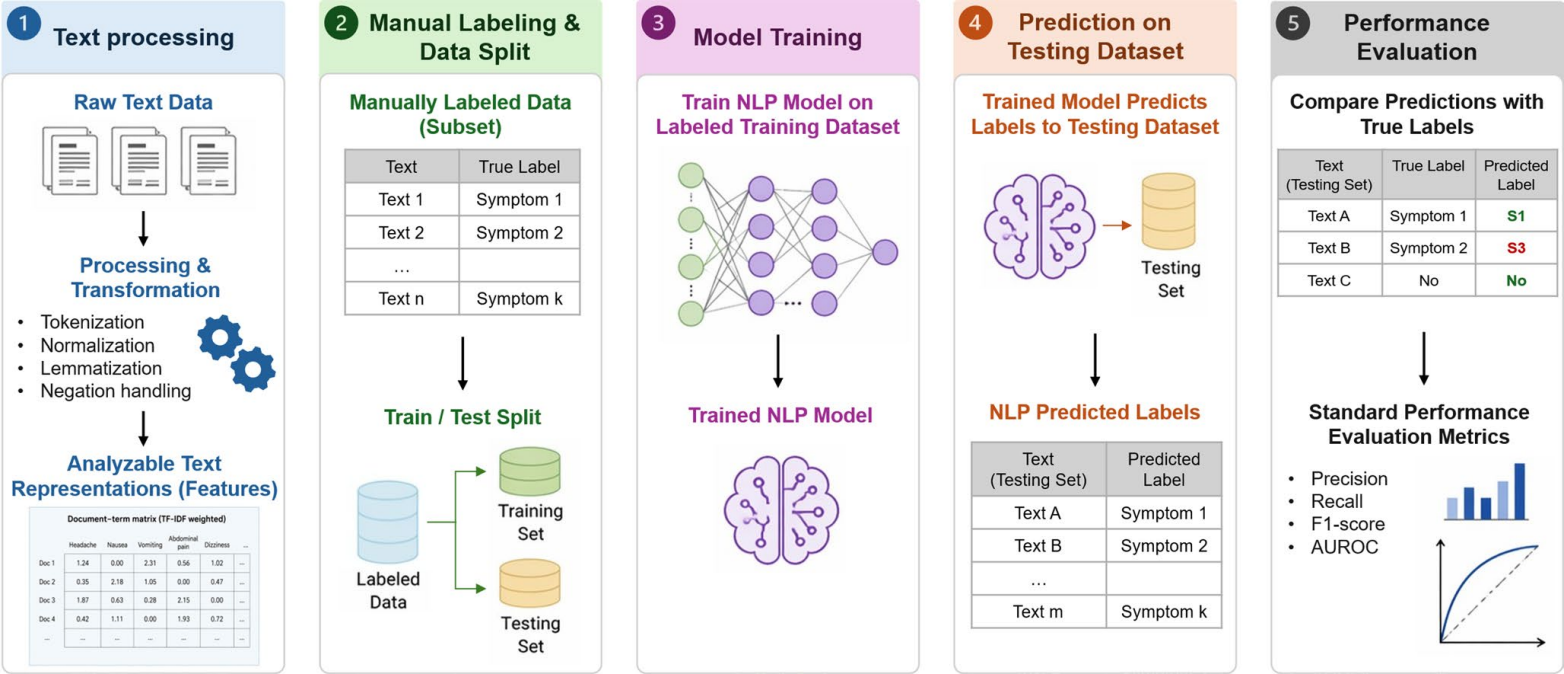
Typical natural language processing text classification pipeline. The text processing techniques listed (e.g., tokenization, normalization, lemmatization, negation handling) are provided as examples and are not exhaustive. For text representation (features), term frequency–inverse document frequency (TF-IDF) is shown as an illustrative example. Alternative methods may also be used. TF-IDF is a commonly used in natural language processing, which converts text into machine-analyzable numerical vectors based on word importance across documents

Modern NLP typically uses computational and machine learning methods to process and analyze human language (Eisenstein, 2019). NLP can be used for various tasks, including text classification, where machines learn patterns from text data and assign a label (e.g., presence or absence of SS) to new text (Eisenstein, 2019). In supervised learning, machines learn from human-labeled data, where the true labels are provided (Nadkarni et al., 2011). A typical NLP text classification pipeline (Fig. 1) includes the following steps: (1) raw text data are processed and transformed into analyzable representations of text (features) using various techniques; (2) a subset of data is manually labeled (true labels) and split into training and testing datasets; (3) an NLP model is trained on the labeled training dataset to learn patterns; (4) the trained NLP model predicts labels for the testing dataset; (5) NLP performance is evaluated by comparing predicted labels with the true labels in the testing dataset.

In the context of HDP, previous studies have used NLP in a varied scope. For example, NLP has been used to extract informative features related to preeclampsia from clinical notes (Sufriyana et al., 2020). Other studies have used NLP to identify cases in which HDP, such as preeclampsia or eclampsia, are mentioned in clinical text (Belsti et al., 2025; Xie et al., 2019). However, NLP has not been used to extract SS of HDP documented in clinical notes. In contrast, NLP has been used to extract SS of other health conditions, such as heart failure and cancer (Koleck et al., 2019), demonstrating a strong potential for similar NLP applications in the context of HDP. Therefore, the primary research question was: How accurately can NLP extract clinically relevant SS of HDP from narrative clinical notes? In this pilot study, we conducted a preliminary evaluation of NLP with the following specific aims: (1) to compare the performance of multiple NLP models in extracting four SS categories (elevated blood pressure, neurological, renal, and hepatic/hematologic); (2) to examine differences in the proportion of patients with HDP diagnoses (gestational hypertension, preeclampsia, and eclampsia) based on ICD-10 codes by SS documentation; and (3) to explore differences in SS documentation by patient race and ethnicity, including a subgroup analysis among patients without any HDP diagnosis.

## Methods

### Data and Study Population

This was a retrospective observational pilot study. We conducted secondary data analyses using EHR data from patients at greater than 20 weeks gestation who were admitted for labor and birth at two urban hospitals in the Northeast United States between 2017 and 2019. Clinical notes used in this study were recorded during the hospital admissions within the same period (2017–2019). Both hospitals used the same EHR platform during this period. We included five note types containing substantive narrative text describing patient assessments: obstetric admission notes, obstetric postpartum notes, obstetric triage notes, anesthesia resident notes, and miscellaneous nursing notes. All patients with at least one note from the five selected note types were considered eligible. We excluded patients without notes from the five selected note types and notes containing no narrative text. The final study sample size included 83,003 clinical notes from 17,775 patients. The dataset did not include repeated admissions for the same patient. There were no multiple admissions within the same pregnancy or more than one birth per patient. Each record represented a single birth event per patient, with a corresponding admission. The average number of notes per patient was 4.7 (see Table S1 in the Supplementary Material). Given the skewed distribution of note counts, we examined the median number of notes by patient race and ethnicity. The median number of notes was three for Hispanic, non-Hispanic American Indian and Alaska Native (AIAN), and non-Hispanic multiracial patients. Non-Hispanic white, non-Hispanic Black, and non-Hispanic Asian Pacific Islander (API) patients had a median of four notes. The current study followed ethical standards and received the Institutional Review Board approval from Columbia University Medical Center (AAAT9870).

## NLP Approach

We implemented the following NLP approach to extract SS. Step 1: We selected four SS categories, informed by clinical guidelines for HDP (ACOG, 2020). We operationally defined each category for annotation purposes to capture SS documentation in real-world clinical notes (Table 1). Thus, these operational definitions may not reflect formal diagnostic criteria of HDP. For example, elevated blood pressure was often described qualitatively without corresponding numeric values in narrative text (e.g., “BP [blood pressure] mildly elevated”). In some cases, numeric values below formal diagnostic thresholds were documented along with qualitative descriptions (e.g., “mildly elevated BPs in the 130s/90s”). These cases were retained to reflect real-world documentation and ensure that annotation captured clinically relevant text. The SS categories and their operational definitions were reviewed by clinical experts in maternal health. Step 2: To create a human-annotated gold standard dataset, we used a high-likelihood sampling approach, where a portion of clinical notes was selected from a subset of patients who were more likely to contain text related to SS. This approach is commonly used in NLP research to improve the efficiency of annotation by prioritizing notes more likely to contain relevant and useful information (Naseem et al., 2021). We identified a subset of patients with any HDP diagnosis based on ICD-10 codes and randomly selected 500 of their clinical notes. We manually annotated these 500 notes for the presence or absence of SS in each of the four categories. Given that this was a pilot study, we annotated a limited number of notes to balance feasibility and efficiency with the goal of a preliminary NLP performance evaluation. To enhance annotation quality, two PhD-prepared nurses (Authors 1 and 2) with clinical backgrounds and research experience in symptom science and health informatics served as reviewers. To ensure consistency, the reviewers participated in a preparatory session prior to the annotation phase, during which they reviewed the operational definitions, discussed example phrases and sentences, and performed practice annotations. The reviewers independently annotated the notes to support reliability. Any disagreement was resolved through discussion until consensus was reached. Step 3: We used the human-annotated gold standard dataset to train and test NLP models. We used five machine learning models (support vector machine [SVM], extreme gradient boosting [XGBoost], random forest, decision tree, and logistic regression), which have been widely used for text classification tasks (Locke et al., 2021). We also used ClinicalBERT (Bidirectional Encoder Representations from Transformers), a pre-trained transformer-based model designed for robust performance in text interpretation (Devlin et al., 2018). We used five-fold cross-validation for training and testing. In five-fold cross-validation, the dataset is randomly divided into five equal subsets (folds). Four subsets are used for training, and one subset is used for testing in each iteration. This process is repeated five times so that each subset is used for testing once. Model performance is averaged across the five iterations. This approach provides more stable and reliable estimates than a single train-test split, as it reduces variability in performance due to how the data are split into training and testing sets (Eisenstein, 2019). We calculated standard evaluation metrics for NLP, including precision, recall, and F1-scores. Precision refers to the proportion of true positives among all positive predictions. Recall refers to the proportion of true positives among all actual positives. F1-score is a harmonic mean of precision and recall and provides a single, balanced metric for evaluating overall performance. Step 4: We applied the final NLP model with the best performance to the full dataset. We calculated and reported descriptive statistics (frequency and percentage) at the note- and patient-levels. All NLP analyses were conducted using Python.

**Table 1:** Categories of signs and symptoms

Category	Operational definition for manual annotation	Examples
Elevated blood pressure	Statements indicating elevated blood pressure, including qualitative descriptions with and without numeric values.	“Sent from perinatal clinic for elevated BPs [blood pressure] in clinic (130s/90s).” “Mild range BPs [blood pressure] noted in triage and pt with a history of chronic HTN [hypertension] controlled with labetalol 100 mg BID [twice a day].”
Neurological SS	Statements indicating SS related to nervous system including headache, vision change, changes in mental status, and seizure.	“Endorsed a short 2-minute HA [headache] self-resolving.” “c/o [complaint of] blurry vision since last night even with eyeglasses.” “States last night she had a presumed seizure.”
Renal SS	Statements indicating SS related to reduced kidney function, including proteinuria or abnormal creatine levels.	“Ruled in for PEC [preeclampsia] without SF [severe features] intrapartum with UPC [urine protein to creatinine ratio] 0.5.” “Transiently elevated Cr [creatinine].” “3 + protein u-dip [urine dipstick].”
Hepatic/hematologic SS	Statements indicating SS related to impaired liver function or blood clotting conditions including thrombocytopenia, hemolysis, epigastric pain, and abnormal liver enzymes.	“36 + 3wks presenting for epigastric pain.” “Labs significant for down trending platelets 99.” “Up trending LFTs [liver function tests].” “PEC [preeclampsia] labs notable for a mild transaminitis to 59/78.”

SS = signs and symptoms

## Statistical Analyses

We conducted descriptive statistics to summarize patient characteristics. We also conducted several exploratory bivariate analyses. First, we examined differences in the proportion of patients with HDP diagnoses (gestational hypertension, preeclampsia, and eclampsia) by the number of SS categories documented, ranging from zero (no documentation in any of the four categories) to four (documentation in all four categories), using a chi-square test. HDP diagnoses were identified using ICD-10 codes recorded in the dataset and were not derived from clinical notes. Second, we examined differences in the proportion of patients with SS documentation by patient race and ethnicity (non-Hispanic white, non-Hispanic Black, Hispanic, and non-Hispanic API) using a chi-square test. Due to small sample sizes, non-Hispanic AIAN and non-Hispanic multiracial patients were excluded from this analysis. Lastly, we conducted a subgroup analysis among patients without ICD-10 codes for HDP (*n* = 14,338). We operationally defined the *NLP-derived SS co-occurrence* as the presence of (1) elevated blood pressure and (2) at least one additional SS category. This reflects the co-occurrences of at least two SS categories, with elevated blood pressure serving as the core indicator. We used a chi-square test to examine whether the proportion of patients with NLP-derived SS co-occurrence differed by patient race and ethnicity. We used SAS 9.4 to conduct all statistical analyses.

## Results

### Description of Patient Characteristics

Patient characteristics of the current study are presented in Table 2. About 69% of the patients were between 20 and 34 years old (*n* = 12,216). Approximately 58% of the patients were Hispanic (*n* = 10,290), 24% were non-Hispanic white (*n* = 4,217), and 12% were non-Hispanic Black (*n* = 2,084). Most patients spoke English (*n* = 10,914; 61.4%). About 55% of the patients were insured by Medicaid (*n* = 9,664; 55.1%). Over half were multiparous (*n* = 8,729; 52.8%). Most patients had vaginal births (*n* = 11,792; 66.3%), and most births occurred at term (*n* = 15,574; 87.6%). About 19% of the patients had at least one HDP diagnosis based on ICD-10 codes (*n* = 3,414).

**Table 2:** Patient characteristics (*N* = 17,775)

	*n*	%
Age group		
≤ 19	662	3.7
20–34	12,216	68.7
≥ 35	4,897	27.6
Race and ethnicity		
Non-Hispanic white	4,217	23.7
Non-Hispanic Black	2,084	11.7
Hispanic	10,290	57.9
Non-Hispanic API	1,160	6.5
Non-Hispanic AIAN	9	0.1
Non-Hispanic multiracial	15	0.1
Preferred spoken language^a^		
English	10,788	61.4
Spanish	5,287	30.1
Other	1,492	8.5
Married^a, b^		
Yes	7,267	41
No	10,469	59
Insurance type^a^		
Medicaid	9,664	55.1
Private	7,867	44.9
Parity^a^		
Nulliparous	7,788	47.2
Multiparous	8,729	52.8
Mode of birth		
Vaginal	11,792	66.3
Cesarean	5,983	33.7
Gestational age at birth		
Term (≥ 37 weeks)	15,574	87.6
Preterm (< 37 weeks)	2,201	12.4
Any HDP diagnosis^c^		
Yes	3,414	19.2
No	14,361	80.8

AIAN = American Indian and Alaska Native, API = Asian Pacific Islander, HDP = hypertensive disorders in pregnancy

^a^ Sample sizes vary due to missing data for the following variables: preferred spoken language (*n* = 17,567; missing = 208, 1.2%), married (*n* = 17,736; missing = 39, 0.2%), insurance type (*n* = 17,531; missing = 244, 1.4%), and parity (*n* = 16,517; missing = 1,258; 7.1%). Percentages are calculated based on non‑missing observations

^b^ Not married includes single, divorced, widowed, unspecified, or unknown

^c^ HDP diagnoses include gestational hypertension, preeclampsia, and eclampsia based on ICD-10 codes, which are not mutually exclusive

### Description of Human Annotation

The agreement rate between the two reviewers was 85.4%, with Cohen’s kappa of 0.71, indicating substantial agreement (Viera & Garrett, 2005). We annotated 363 examples of SS (text fragments within clinical notes). Among these examples, elevated blood pressure was most frequently documented (*n* = 248, 68.3%), followed by neurological SS (*n* = 45, 12.4%) and renal SS (*n* = 45, 12.4%). Hepatic/hematologic SS (*n* = 25, 6.9%) were less frequently documented.

## NLP Model Performance

NLP model performance by SS category and the macro-average across all SS categories is presented in Table 3. XGBoost achieved the highest performance, with the macro-average F1-score of 0.75. Among SS categories, elevated blood pressure demonstrated the highest performance, with F1-scores ranging from 0.80 (Decision Tree and ClinicalBERT) to 0.87 (XGBoost) across different models. This was followed by neurological SS, with F1-scores ranging from 0.65 (ClinicalBERT) to 0.77 (XGBoost). Renal and hepatic/hematologic SS showed lower F1-scores.

**Table 3:** Natural language processing model performance (*N* = 500 clinical notes)

SS categories	Precision	Recall	F1-score	Precision	Recall	F1-score
	XGBoost			SVM		
Elevated blood pressure	0.87	0.87	0.87	0.84	0.83	0.83
Neurological SS	0.92	0.73	0.77	0.86	0.72	0.75
Renal SS	0.64	0.69	0.65	0.63	0.72	0.62
Hepatic/hematologic SS	0.78	0.70	0.70	0.73	0.63	0.64
Macro-average	0.80	0.75	0.75	0.77	0.73	0.71
	Random forest			Decision tree		
Elevated blood pressure	0.85	0.85	0.85	0.80	0.80	0.80
Neurological SS	0.77	0.69	0.72	0.69	0.67	0.67
Renal SS	0.65	0.69	0.65	0.60	0.61	0.60
Hepatic/hematologic SS	0.78	0.71	0.70	0.67	0.64	0.65
Macro-average	0.76	0.74	0.73	0.69	0.68	0.68
	Logistic regression			ClinicalBERT		
Elevated blood pressure	0.83	0.82	0.82	0.80	0.80	0.80
Neurological SS	0.77	0.74	0.74	0.80	0.61	0.65
Renal SS	0.63	0.73	0.63	0.62	0.63	0.62
Hepatic/hematologic SS	0.68	0.65	0.65	0.47	0.50	0.49
Macro-average	0.73	0.74	0.71	0.67	0.64	0.64

BERT = Bidirectional Encoder Representations from Transformers, SS = signs and symptoms, SVM = support vector machine, XGBoost = extreme gradient boosting

## Application of the Best-Performing Model

We applied the best-performing model, XGBoost, to the full dataset (*N* = 83,003 notes; Table 4). About 1 in 4 notes had at least one SS category documented (*n* = 20,167; 24.3%). Elevated blood pressure was most frequently documented (*n* = 18,935; 22.8%), followed by renal SS (*n* = 4,983; 6%). At the patient-level (*N* = 17,775), 42.3% of patients (*n* = 7,520) had at least one SS category documented. Similar to the note-level, elevated blood pressure was most frequently documented (*n* = 5,587; 31.4%), followed by renal SS (*n* = 3,636; 20.5%).

**Table 4:** Application of XGBoost to the full dataset

SS categories^a^	*n*	%
	Note-level *N* = 83,003	
Any SS	20,167	24.3
Elevated blood pressure	18,935	22.8
Neurological SS	778	0.9
Renal SS	4,983	6.0
Hepatic/hematologic SS	1,810	2.2
	Patient-level *N* = 17,775	
Any SS	7,520	42.3
Elevated blood pressure (two or more notes)	5,587	31.4
Neurological SS	664	3.7
Renal SS	3,636	20.5
Hepatic/hematologic SS	1,528	8.6

SS = signs and symptoms, XGBoost = extreme gradient boosting

^a^ SS categories are not mutually exclusive

### HDP Diagnoses by SS Documentation

**Fig. 2:**
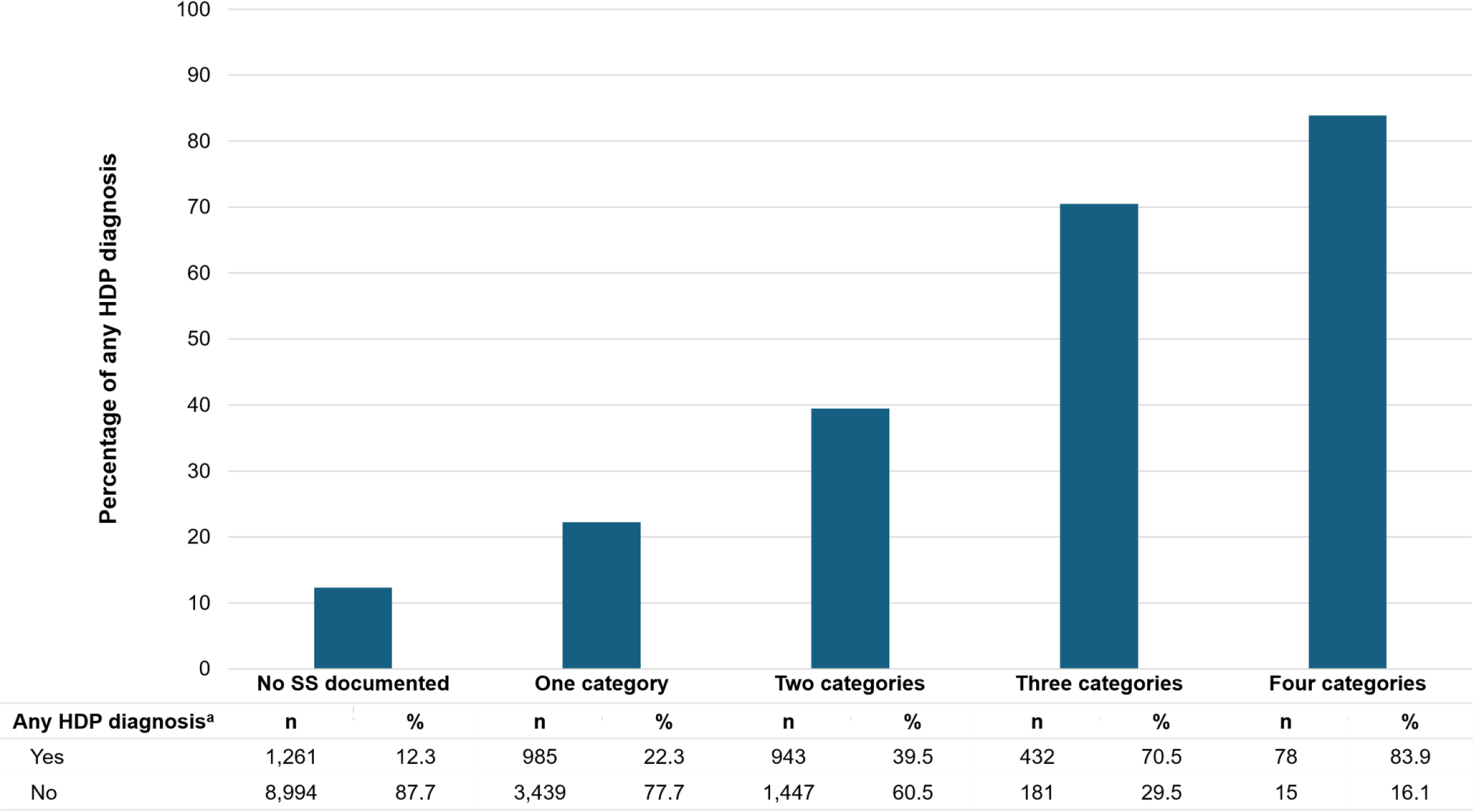
Proportion of hypertensive disorders in pregnancy diagnoses by the number of signs and symptoms documented (*N* = 17,775). HDP = hypertensive disorders in pregnancy, SS = signs and symptoms. ^a^ HDP diagnoses include gestational hypertension, preeclampsia, and eclampsia based on ICD-10 codes

The proportion of patients with HDP diagnoses (based on ICD-10 codes) differed significantly by the number of SS categories documented (*p* < .001, Fig. 2). We observed a higher proportion of HDP diagnoses as the number of documented SS categories increased. Among patients with SS documented in all four categories, 83.9% had an HDP diagnosis (*n* = 78). In contrast, among those without any documented SS, 12.3% had an HDP diagnosis (*n* = 1,261).

### SS Documentation by Patient Race and Ethnicity

**Fig. 3:**
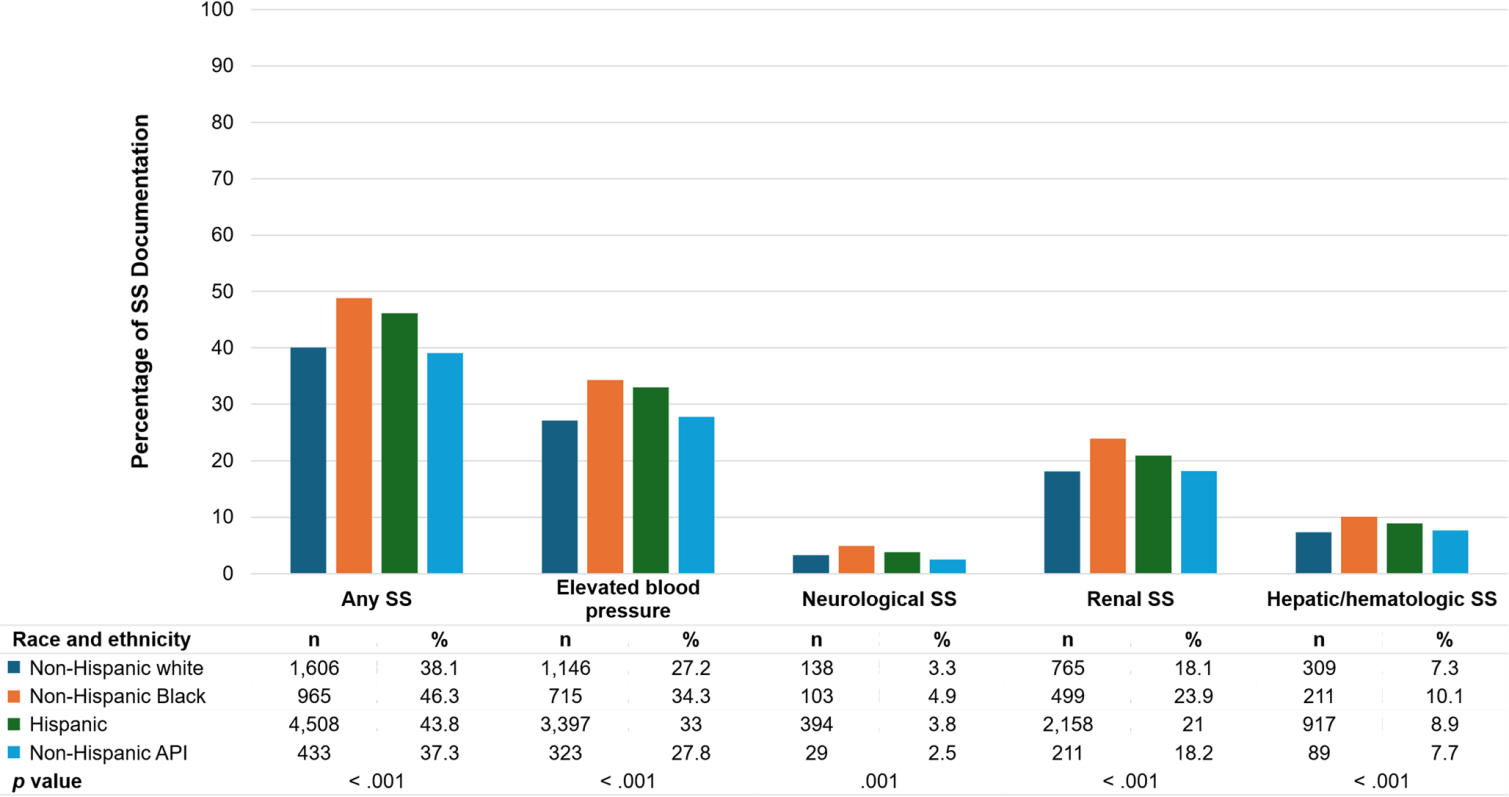
Documentation of signs and symptoms by patient race and ethnicity (*N* = 17,751) . API = Asian Pacific Islander, SS = signs and symptoms

The proportion of patients with SS documentation differed significantly by patient race and ethnicity (Fig. 3). A higher proportion of non-Hispanic Black (*n* = 965, 46.3%) and Hispanic (*n* = 4,508; 43.8%) patients had documentation of at least one SS category than non-Hispanic white (*n* = 1,606; 38.1%) or non-Hispanic API (*n* = 433, 37.3%) patients (*p* < .001). Similarly, a higher proportion of non-Hispanic Black patients had SS documentation across all categories (*p* = .001 for neurological SS and *p* < .001 for the remaining categories).

**Fig. 4:**
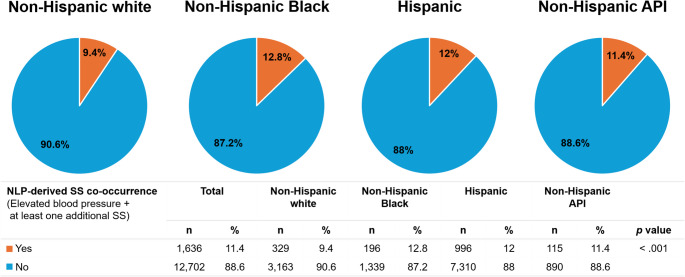
Proportion of patients with NLP-derived signs and symptoms co-occurrence among those without hypertensive disorders in pregnancy diagnosis (Based on ICD-10 Codes) by patient race and ethnicity (*N* = 14,338). API = Asian Pacific Islander, SS = signs and symptoms

In a subgroup analysis among patients without ICD-10 codes for HDP (*n* = 14,338), the proportion of patients with NLP-derived SS co-occurrence differed significantly by patient race and ethnicity (Fig. 4). Overall, approximately 11% of patients without ICD-10 codes for HDP had NLP-derived SS co-occurrence (*n* = 1,636). A higher proportion of non-Hispanic Black (*n* = 196, 12.8%), Hispanic (*n* = 996, 12%), and non-Hispanic API (*n* = 115, 11.4%) patients had NLP-derived SS co-occurrence than non-Hispanic white patients (*n* = 329, 9.4%; *p* < .001).

## Discussion

In this pilot study, we evaluated the performance of NLP models in extracting clinically relevant SS of HDP from narrative clinical notes. This study extends prior work by using NLP to extract SS from clinical notes, rather than focusing on general features or explicit mentions of diagnosis, providing a more granular examination of SS documentation. Overall, the findings suggest that NLP can extract SS with moderate accuracy, supporting methodological feasibility for larger-scale extraction. Among the various models evaluated, XGBoost demonstrated the highest overall performance, achieving the macro-average F1-score of 0.75. XGBoost also outperformed other models across individual SS categories. Several factors may explain this finding. XGBoost is a feature-based machine learning model, making it more advantageous in identifying SS, which often appear as explicit or straightforward keywords and phrases in clinical notes. In contrast, ClinicalBERT is text-embedding-based, which is better at understanding and capturing nuanced context and linguistic patterns (Devlin et al., 2018). Additionally, XGBoost may be better suited to smaller labeled datasets than ClinicalBERT, which typically requires larger labeled datasets to achieve optimal performance (Lamproudis et al., 2022; Zou et al., 2022).

Applying XGBoost to the full dataset revealed that approximately one in four clinical notes and 42% of patients had at least one SS category documented. Elevated blood pressure was the most frequently documented, followed by renal SS at both the note and patient-levels. It is important to note that although SS categories were conceptually distinct, several SS included in this study are not specific to HDP and may overlap with other conditions. For example, neurological symptoms such as headaches may be related to dehydration or sleep deprivation, while elevated blood pressure may reflect pain, anxiety, or acute stress. Thus, findings should be interpreted with caution, as SS documentation does not necessarily indicate HDP. In particular, although approximately 11% of patients without ICD-10 codes for HDP had NLP-derived SS co-occurrence, this does not necessarily indicate undiagnosed HDP. To partially account for this non-specificity, we defined NLP-derived SS co-occurrence as the presence of elevated blood pressure, serving as the core indicator, and at least one additional SS. Despite the potential non-specificity of SS to HDP, progressively higher proportions of HDP diagnoses were observed as the number of documented SS categories increased. This pattern supports an association between SS documentation and HDP diagnoses. It is also consistent with the expectation that HDP diagnoses are more commonly observed in the presence of multiple co-occurring SS.

Non-Hispanic Black patients consistently had the highest proportion of documented SS overall and for all categories. This finding likely reflects well-established disparities, as non-Hispanic Black patients have a higher prevalence of HDP (Ford et al., 2022). In a subgroup analysis of patients without ICD-10 codes for HDP, a higher proportion of non-Hispanic Black, Hispanic, and non-Hispanic API patients had NLP-derived SS co-occurrence without any HDP diagnosis than non-Hispanic white patients. These findings should be interpreted cautiously, as SS documentation does not necessarily indicate HDP. However, this may reflect differences in documentation practices and HDP recognition among racially and ethnically diverse patients. Previous research has shown that hypertensive disorders are often underdiagnosed and underreported among Black women and Hispanic individuals in general (Britton et al., 2018; Castaneda et al., 2020). Future research is needed to better understand these differences and their implications for disparities in HDP recognition and management.

Clinically, SS documentation may reflect multiple aspects of care. Prior research has suggested that documentation of elevated blood pressure in clinical notes may serve as a marker of clinical recognition (Chapman et al., 2025). Patients with text related to elevated blood pressure documented in their clinical notes had shorter delays in hypertension diagnosis (Chapman et al., 2026). Similar patterns have been observed in other conditions. For example, documentation of obesity in the problem list has been associated with clinicians addressing obesity in future visits (Banerjee et al., 2013). At the same time, absence of documentation does not necessarily indicate the absence of SS, as documentation may be influenced by competing clinical priorities and clinicians’ limited time. In this context, our findings demonstrate that SS can be systematically extracted from clinical notes to enable the examination of documentation patterns at a larger scale. Future research is needed to better understand how SS documentation relates to clinical workflow, recognition, and management of HDP.

Our findings also highlight the value of leveraging narrative clinical notes, where approximately 80% of health information is stored within EHRs, for SS extraction (Li et al., 2022). The diagnostic criteria for HDP primarily rely on objective measures, such as elevated blood pressure, elevated protein/creatinine ratio, and/or low platelet counts (ACOG, 2020), which are typically recorded in structured fields within EHRs (Grunewald et al., 2025). Previous research has indicated that structured EHR data are often incomplete or inconsistently recorded, with missingness in vital signs and laboratory values (Senthinathan et al., 2023; Sun et al., 2024). Thus, our findings provide preliminary evidence supporting the use of NLP as a complementary approach to capture clinically relevant SS that may not be consistently represented in structured EHR data, potentially enhancing the completeness of clinical information for EHR-based research and population-level surveillance. In addition, NLP can be integrated into EHRs to facilitate clinicians in navigating complex data by aggregating SS documented across multiple notes to support more efficient review of SS (Hackeloer et al., 2023). Implementing NLP into clinical practice for SS extraction will require further research and development. This includes integration within EHR systems for real-time processing of notes, alignment with existing clinical workflow without increasing clinicians’ burden, and validation across diverse health care systems to ensure reliability.

### Limitations

This study has several important limitations. First, information on the exact timing of HDP diagnoses relative to note documentation was not available in the dataset. Therefore, we were unable to determine whether the notes were documented before, at the time of, or after diagnosis. Given this limitation, findings should not be interpreted as evidence that SS documentation has predictive value for diagnosis. Rather, this pilot study represents an initial step in evaluating how accurately NLP can extract various SS from clinical notes. Future research is needed to examine whether SS documented prior to diagnosis can improve prediction of HDP. Second, the sample size of our annotated dataset was small (*n* = 500 clinical notes). This may limit performance, particularly for less prevalent SS. For example, we observed lower F1-scores for renal and hepatic/hematologic SS, which may be attributable to the limited number of annotated examples for these categories. In addition, models trained on limited annotated data may be sensitive to sampling variability and may not generalize well to new data. We employed five-fold cross-validation, which can provide more stable estimates of performance when annotated data are limited (Eisenstein, 2019). NLP often requires substantial high-quality human-annotated data, which are highly resource-intensive to produce in terms of time and human labor (Frei & Kramer, 2023). Due to the preliminary nature of this pilot study and resource constraints, additional annotation was not feasible. Future work should expand annotated data or consider data augmentation techniques, such as synthetic data generation, to improve performance.

Third, inherent variability in clinical documentation may affect the reliability and validity of text data obtained from clinical notes. For example, despite employing various preprocessing steps, separating structured and unstructured fields of clinical notes remains a challenge, which may introduce noise or omit relevant text. Clinical notes may also incompletely capture all SS experienced by patients, as documentation depends on multiple factors (e.g., clinician assessment, patient report, and documentation practice), which may affect the validity of text data. Thus, absence of documentation should not be interpreted as the true absence of clinically relevant SS. In addition, while the specific EHR platform used at both hospitals did not change during the period when clinical notes were recorded, we did not have information on potential major changes within the EHR platform, which may have influenced documentation practices. Fourth, NLP-derived SS co-occurrence was defined based on common SS of HDP in general (i.e., elevated blood pressure and at least one additional SS) and should not be interpreted as formal diagnostic criteria. Fifth, the race and ethnicity variable was limited to categories available in the dataset and could not be further specified. Future studies should incorporate more granular categorization to improve the specificity of findings and provide additional insights. For example, differences in the proportion of documented SS may exist within Hispanic populations (e.g., Black Hispanic vs. white Hispanic). This is important to consider as prior research has shown that Black Hispanic patients have higher odds of HDP than non-Black Hispanic patients (Atkinson et al., 2026). Similarly, we did not have information on how communication occurred for non-English speaking patients during clinical encounters (e.g., use of interpreter services), which may influence how SS are communicated and documented. Lastly, our study was conducted within two urban hospitals in the Northeast United States, which may limit generalizability to other geographic locations and settings. Specifically, the prevalence of HDP based on ICD-10 codes in our sample (approximately 19%) was higher than the population-level prevalence (approximately 16%). A higher proportion of patients with a formal diagnosis may have contributed to increased SS documentation overall. Therefore, findings may not be generalizable to settings with lower prevalence. Future studies should consider validating our approach in diverse clinical settings to ensure broader applicability.

## Conclusions

In this pilot study, NLP demonstrated moderate performance in extracting clinically relevant SS of HDP from narrative clinical notes, supporting feasibility for large-scale extraction. Our findings provide preliminary evidence supporting the use of NLP as a complementary approach to capture SS that may not be consistently represented in structured EHR data. In addition, these findings offer insights into potential differences in SS documentation by race and ethnicity. Future research is needed to improve NLP performance, including expanding annotated data for training and testing and validating findings across diverse settings to enhance generalizability.

## Supplementary Information

Below is the link to the electronic supplementary material.

Supplementary Material 1

## Data Availability

No data is available for public access due to the limitations imposed by the Institutional Review Board protocol.
