# Supplementary material for: Extracting Signs and Symptoms of Hypertensive Disorders in Pregnancy from Clinical Notes Using Natural Language Processing
Source: Matern Child Health J. 2026 May 20;30(6):809–19. doi: 10.1007/s10995-026-04275-y (PMC13275635; doi:10.1007/s10995-026-04275-y)
Supplement: Supplementary file 1 — Supplementary Material 1 [file 10995_2026_4275_MOESM1_ESM.docx]

**Supplementary Materials**

**Table S1.** Descriptive Statistics of Note Counts

|  | **Number of patients** | **Number of notes** | **Mean number of notes** | **Standard deviation** | **Median number of notes** | **Interquartile range** |
| --- | --- | --- | --- | --- | --- | --- |
| Total | 17,775 | 83,003 | 4.67 | 6.32 | 3 | 3 |
| Race and ethnicity^a^ |  |  |  |  |  |  |
| Non-Hispanic white | 4,217 | 23,295 | 5.52 | 8.62 | 4 | 3 |
| Non-Hispanic Black | 2,084 | 11,842 | 5.68 | 7.41 | 4 | 3 |
| Hispanic | 10,290 | 41,797 | 4.06 | 4.59 | 3 | 3 |
| Non-Hispanic API | 1,160 | 5,868 | 5.06 | 6.62 | 4 | 2 |
| Non-Hispanic AIAN | 9 | 53 | 5.89 | 5.90 | 3 | 3 |
| Non-Hispanic Multiracial | 15 | 148 | 9.87 | 18.57 | 3 | 3 |

*Note.* AIAN = American Indian and Alaska Native, API = Asian Pacific Islander.
